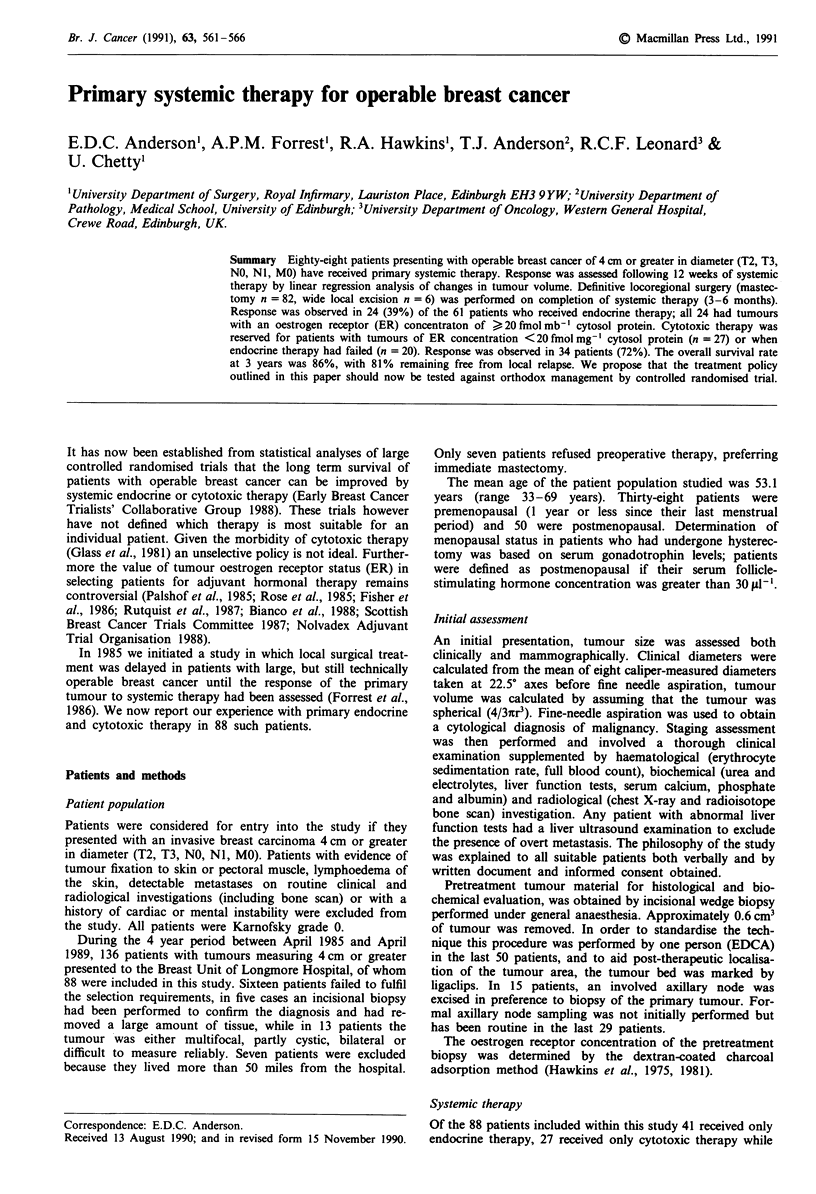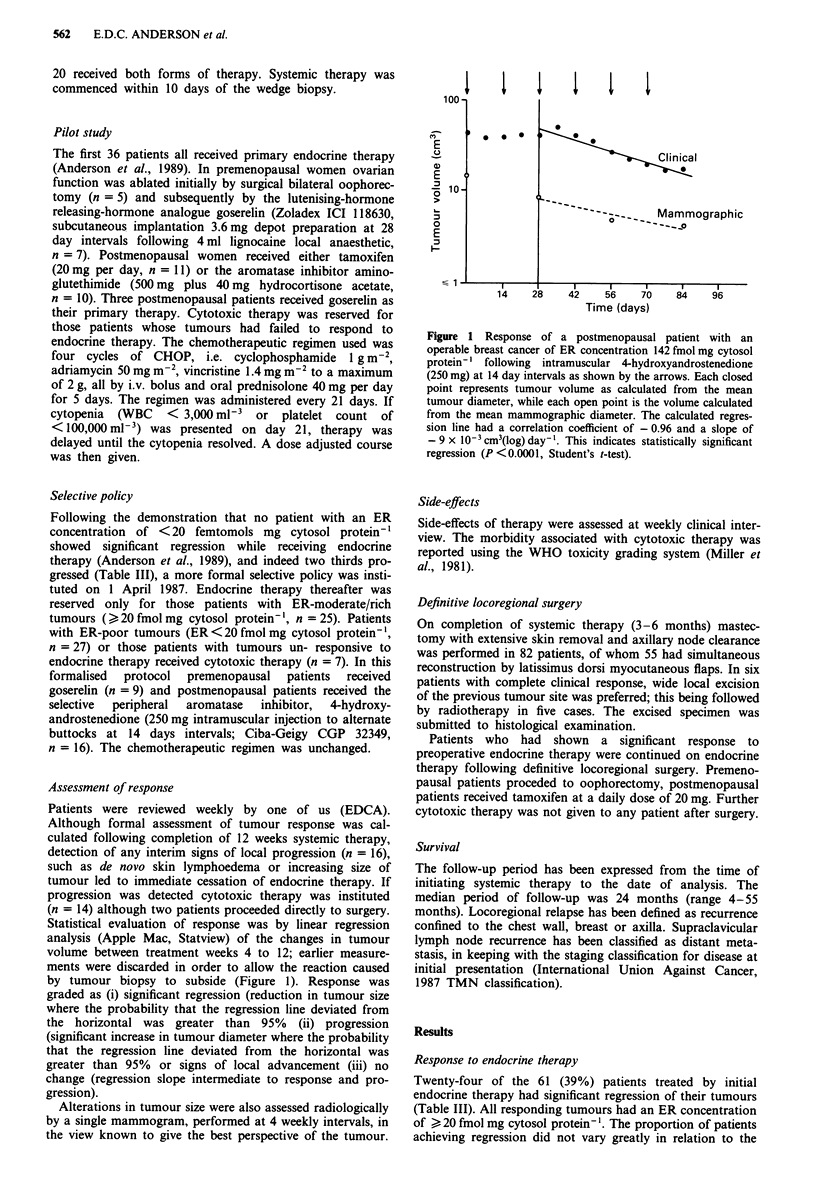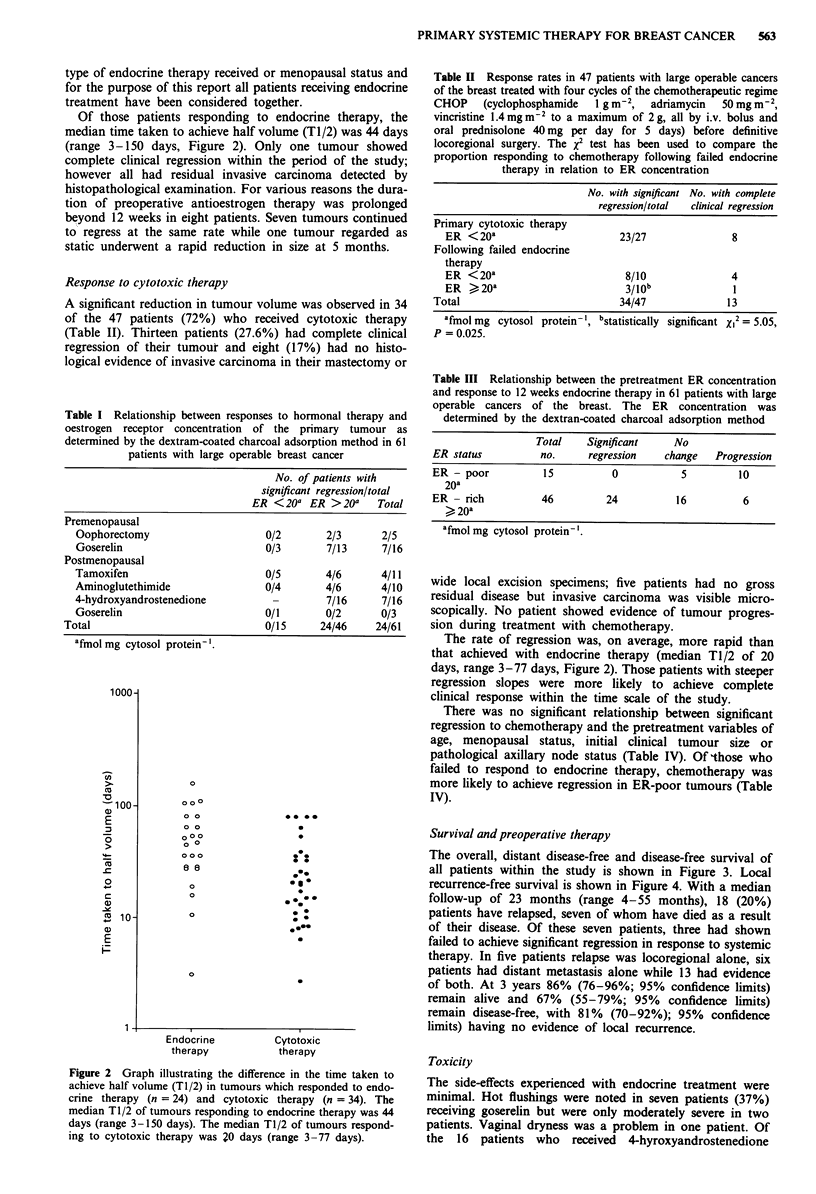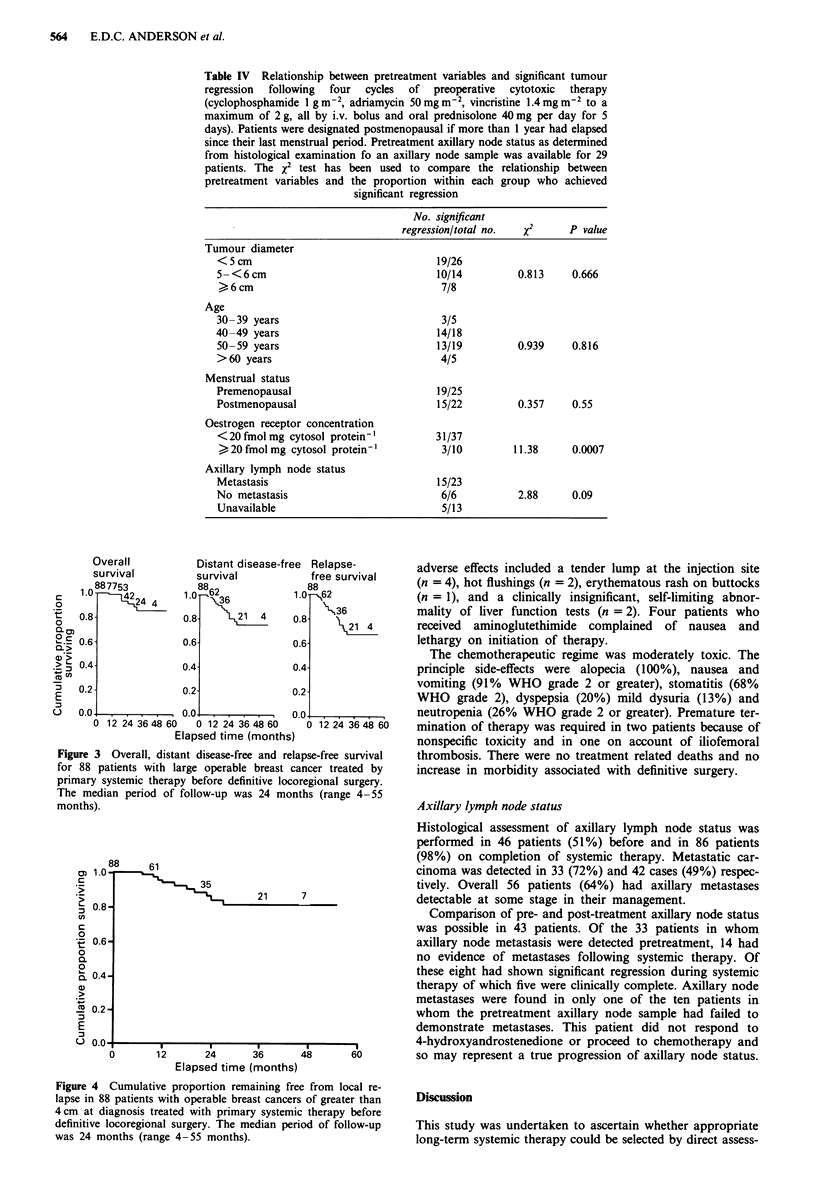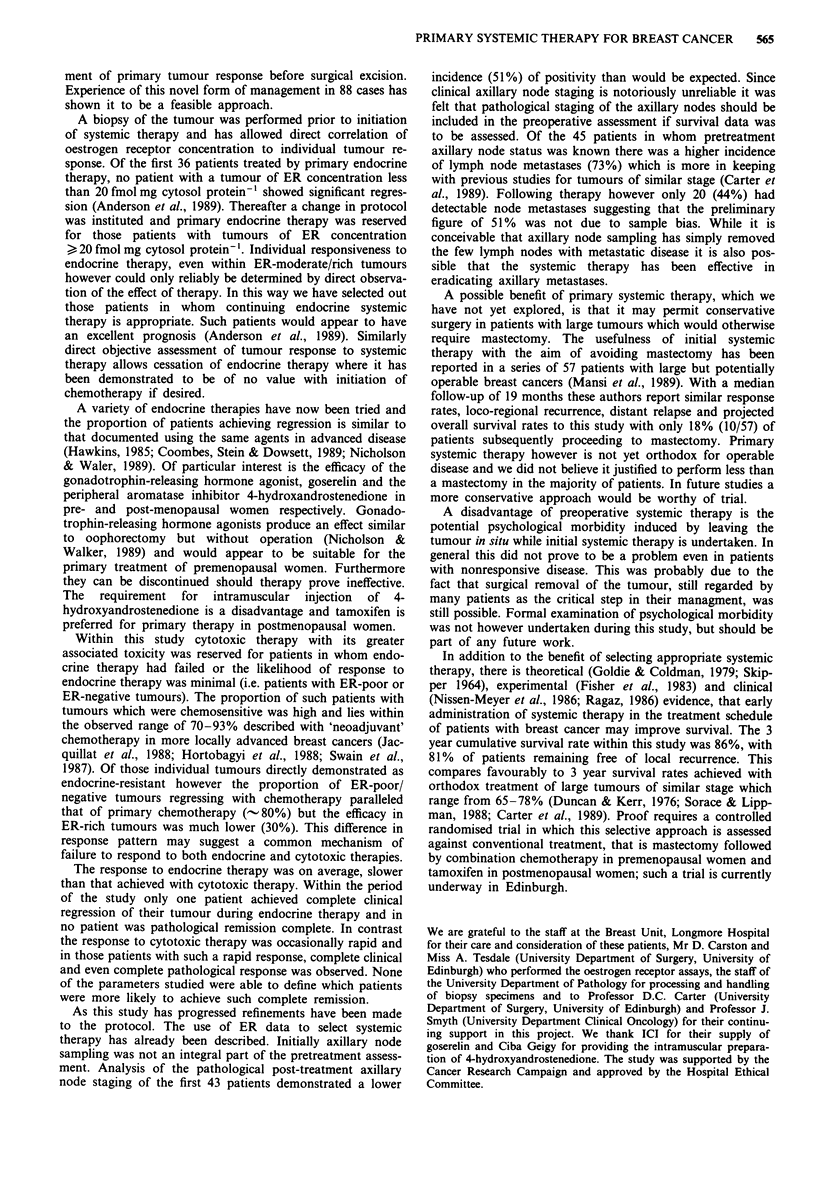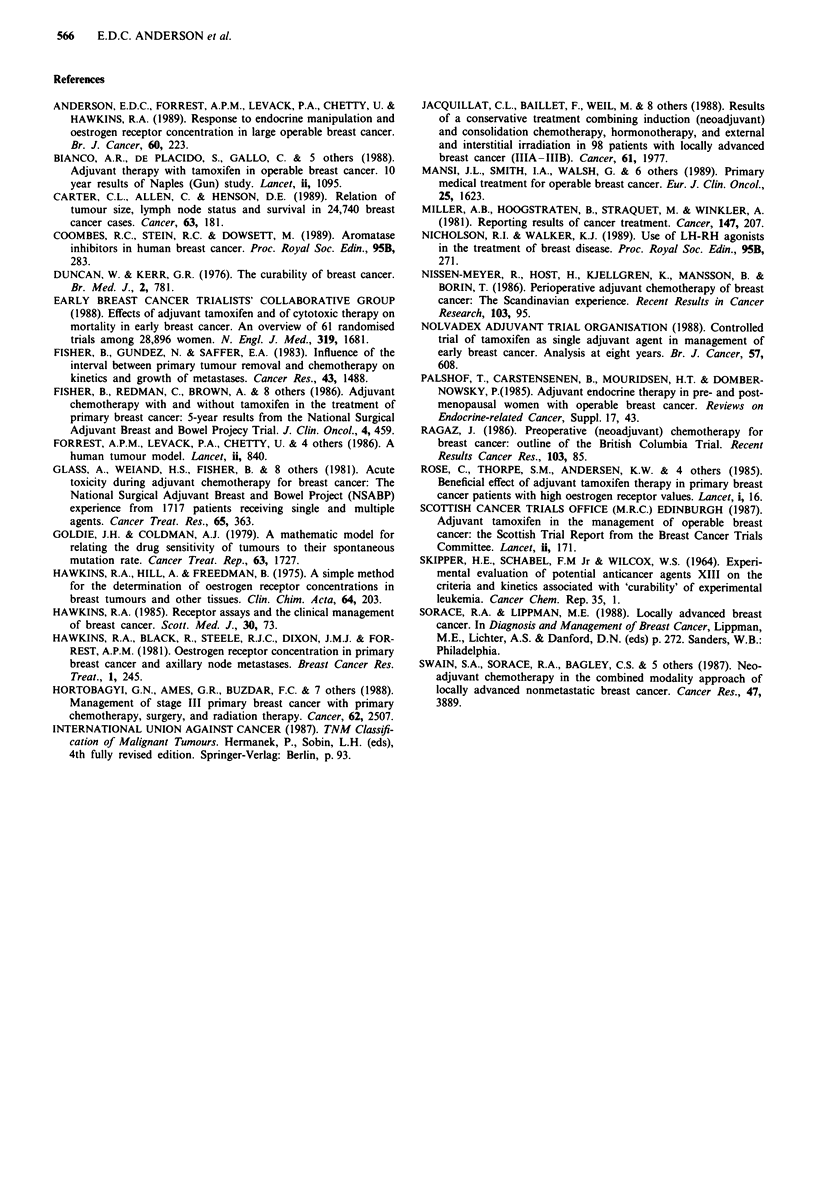# Primary systemic therapy for operable breast cancer.

**DOI:** 10.1038/bjc.1991.131

**Published:** 1991-04

**Authors:** E. D. Anderson, A. P. Forrest, R. A. Hawkins, T. J. Anderson, R. C. Leonard, U. Chetty

**Affiliations:** University Department of Surgery, Royal Infirmary, Edinburgh, UK.

## Abstract

Eighty-eight patients presenting with operable breast cancer of 4 cm or greater in diameter (T2, T3, N0, N1, M0) have received primary systemic therapy. Response was assessed following 12 weeks of systemic therapy by linear regression analysis of changes in tumour volume. Definitive locoregional surgery (mastectomy n = 82, wide local excision n = 6) was performed on completion of systemic therapy (3-6 months). Response was observed in 24 (39%) of the 61 patients who received endocrine therapy; all 24 had tumours with an oestrogen receptor (ER) concentration of greater than or equal to 20 fmol mb-1 cytosol protein. Cytotoxic therapy was reserved for patients with tumours of ER concentration less than 20 fmol mg-1 cytosol protein (n = 27) or when endocrine therapy had failed (n = 20). Response was observed in 34 patients (72%). The overall survival rate at 3 years was 86%, with 81% remaining free from local relapse. We propose that the treatment policy outlined in this paper should now be tested against orthodox management by controlled randomised trial.


Br. J. Cancer (1991), 63, 561–566 © Macmillan Press Ltd., 1991

### Primary systemic therapy for operable breast cancer

E.D.C. Anderson[1], A.P.M. Forrest[1], R.A. Hawkins[1], T.J. Anderson[2], R.C.F. Leonard[3] & U. Chetty[1]

[1]*University Department of Surgery, Royal Infirmary, Lauriston Place, Edinburgh EH3 9YW;* [2]*University Department of Pathology, Medical School, University of Edinburgh;* [3]*University Department of Oncology, Western General Hospital, Crewe Road, Edinburgh, UK.*

**Summary** Eighty-eight patients presenting with operable breast cancer of 4 cm or greater in diameter (T2, T3, N0, N1, M0) have received primary systemic therapy. Response was assessed following 12 weeks of systemic therapy by linear regression analysis of changes in tumour volume. Definitive locoregional surgery (mastectomy $n = 82$, wide local excision $n = 6$) was performed on completion of systemic therapy (3–6 months). Response was observed in 24 (39%) of the 61 patients who received endocrine therapy; all 24 had tumours with an oestrogen receptor (ER) concentraton of $\geqslant 20$ fmol mb$^{-1}$ cytosol protein. Cytotoxic therapy was reserved for patients with tumours of ER concentration $<20$ fmol mg$^{-1}$ cytosol protein ($n = 27$) or when endocrine therapy had failed ($n = 20$). Response was observed in 34 patients (72%). The overall survival rate at 3 years was 86%, with 81% remaining free from local relapse. We propose that the treatment policy outlined in this paper should now be tested against orthodox management by controlled randomised trial.

It has now been established from statistical analyses of large controlled randomised trials that the long term survival of patients with operable breast cancer can be improved by systemic endocrine or cytotoxic therapy (Early Breast Cancer Trialists' Collaborative Group 1988). These trials however have not defined which therapy is most suitable for an individual patient. Given the morbidity of cytotoxic therapy (Glass *et al.*, 1981) an unselective policy is not ideal. Furthermore the value of tumour oestrogen receptor status (ER) in selecting patients for adjuvant hormonal therapy remains controversial (Palshof *et al.*, 1985; Rose *et al.*, 1985; Fisher *et al.*, 1986; Rutquist *et al.*, 1987; Bianco *et al.*, 1988; Scottish Breast Cancer Trials Committee 1987; Nolvadex Adjuvant Trial Organisation 1988).

In 1985 we initiated a study in which local surgical treatment was delayed in patients with large, but still technically operable breast cancer until the response of the primary tumour to systemic therapy had been assessed (Forrest *et al.*, 1986). We now report our experience with primary endocrine and cytotoxic therapy in 88 such patients.

### Patients and methods

### *Patient population*

Patients were considered for entry into the study if they presented with an invasive breast carcinoma 4 cm or greater in diameter (T2, T3, N0, N1, M0). Patients with evidence of tumour fixation to skin or pectoral muscle, lymphoedema of the skin, detectable metastases on routine clinical and radiological investigations (including bone scan) or with a history of cardiac or mental instability were excluded from the study. All patients were Karnofsky grade 0.

During the 4 year period between April 1985 and April 1989, 136 patients with tumours measuring 4 cm or greater presented to the Breast Unit of Longmore Hospital, of whom 88 were included in this study. Sixteen patients failed to fulfil the selection requirements, in five cases an incisional biopsy had been performed to confirm the diagnosis and had removed a large amount of tissue, while in 13 patients the tumour was either multifocal, partly cystic, bilateral or difficult to measure reliably. Seven patients were excluded because they lived more than 50 miles from the hospital. Only seven patients refused preoperative therapy, preferring immediate mastectomy.

The mean age of the patient population studied was 53.1 years (range 33–69 years). Thirty-eight patients were premenopausal (1 year or less since their last menstrual period) and 50 were postmenopausal. Determination of menopausal status in patients who had undergone hysterectomy was based on serum gonadotrophin levels; patients were defined as postmenopausal if their serum follicle-stimulating hormone concentration was greater than 30 $\mu l^{-1}$.

### *Initial assessment*

An initial presentation, tumour size was assessed both clinically and mammographically. Clinical diameters were calculated from the mean of eight caliper-measured diameters taken at 22.5° axes before fine needle aspiration, tumour volume was calculated by assuming that the tumour was spherical ($4/3\pi r^3$). Fine-needle aspiration was used to obtain a cytological diagnosis of malignancy. Staging assessment was then performed and involved a thorough clinical examination supplemented by haematological (erythrocyte sedimentation rate, full blood count), biochemical (urea and electrolytes, liver function tests, serum calcium, phosphate and albumin) and radiological (chest X-ray and radioisotope bone scan) investigation. Any patient with abnormal liver function tests had a liver ultrasound examination to exclude the presence of overt metastasis. The philosophy of the study was explained to all suitable patients both verbally and by written document and informed consent obtained.

Pretreatment tumour material for histological and biochemical evaluation, was obtained by incisional wedge biopsy performed under general anaesthesia. Approximately 0.6 cm$^3$ of tumour was removed. In order to standardise the technique this procedure was performed by one person (EDCA) in the last 50 patients, and to aid post-therapeutic localisation of the tumour area, the tumour bed was marked by ligaclips. In 15 patients, an involved axillary node was excised in preference to biopsy of the primary tumour. Formal axillary node sampling was not initially performed but has been routine in the last 29 patients.

The oestrogen receptor concentration of the pretreatment biopsy was determined by the dextran-coated charcoal adsorption method (Hawkins *et al.*, 1975, 1981).

### *Systemic therapy*

Of the 88 patients included within this study 41 received only endocrine therapy, 27 received only cytotoxic therapy while

Correspondence: E.D.C. Anderson.
Received 13 August 1990; and in revised form 15 November 1990.

20 received both forms of therapy. Systemic therapy was commenced within 10 days of the wedge biopsy.

*Pilot study*

The first 36 patients all received primary endocrine therapy (Anderson *et al.*, 1989). In premenopausal women ovarian function was ablated initially by surgical bilateral oophorectomy ($n = 5$) and subsequently by the lutenising-hormone releasing-hormone analogue goserelin (Zoladex ICI 118630, subcutaneous implantation 3.6 mg depot preparation at 28 day intervals following 4 ml lignocaine local anaesthetic, $n = 7$). Postmenopausal women received either tamoxifen (20 mg per day, $n = 11$) or the aromatase inhibitor aminoglutethimide (500 mg plus 40 mg hydrocortisone acetate, $n = 10$). Three postmenopausal patients received goserelin as their primary therapy. Cytotoxic therapy was reserved for those patients whose tumours had failed to respond to endocrine therapy. The chemotherapeutic regimen used was four cycles of CHOP, i.e. cyclophosphamide 1 g $m^{-2}$, adriamycin 50 mg $m^{-2}$, vincristine 1.4 mg $m^{-2}$ to a maximum of 2 g, all by i.v. bolus and oral prednisolone 40 mg per day for 5 days. The regimen was administered every 21 days. If cytopenia (WBC $< 3,000\,ml^{-3}$ or platelet count of $< 100,000\,ml^{-3}$) was presented on day 21, therapy was delayed until the cytopenia resolved. A dose adjusted course was then given.

*Selective policy*

Following the demonstration that no patient with an ER concentration of <20 femtomols mg cytosol protein$^{-1}$ showed significant regression while receiving endocrine therapy (Anderson *et al.*, 1989), and indeed two thirds progressed (Table III), a more formal selective policy was instituted on 1 April 1987. Endocrine therapy thereafter was reserved only for those patients with ER-moderate/rich tumours ($\geqslant$20 fmol mg cytosol protein$^{-1}$, $n = 25$). Patients with ER-poor tumours (ER<20 fmol mg cytosol protein$^{-1}$, $n = 27$) or those patients with tumours un- responsive to endocrine therapy received cytotoxic therapy ($n = 7$). In this formalised protocol premenopausal patients received goserelin ($n = 9$) and postmenopausal patients received the selective peripheral aromatase inhibitor, 4-hydroxy-androstenedione (250 mg intramuscular injection to alternate buttocks at 14 days intervals; Ciba-Geigy CGP 32349, $n = 16$). The chemotherapeutic regimen was unchanged.

*Assessment of response*

Patients were reviewed weekly by one of us (EDCA). Although formal assessment of tumour response was calculated following completion of 12 weeks systemic therapy, detection of any interim signs of local progression ($n = 16$), such as *de novo* skin lymphoedema or increasing size of tumour led to immediate cessation of endocrine therapy. If progression was detected cytotoxic therapy was instituted ($n = 14$) although two patients proceeded directly to surgery. Statistical evaluation of response was by linear regression analysis (Apple Mac, Statview) of the changes in tumour volume between treatment weeks 4 to 12; earlier measurements were discarded in order to allow the reaction caused by tumour biopsy to subside (Figure 1). Response was graded as (i) significant regression (reduction in tumour size where the probability that the regression line deviated from the horizontal was greater than 95% (ii) progression (significant increase in tumour diameter where the probability that the regression line deviated from the horizontal was greater than 95% or signs of local advancement (iii) no change (regression slope intermediate to response and progression).

Alterations in tumour size were also assessed radiologically by a single mammogram, performed at 4 weekly intervals, in the view known to give the best perspective of the tumour.

**Figure 1** Response of a postmenopausal patient with an operable breast cancer of ER concentration 142 fmol mg cytosol protein$^{-1}$ following intramuscular 4-hydroxyandrostenedione (250 mg) at 14 day intervals as shown by the arrows. Each closed point represents tumour volume as calculated from the mean tumour diameter, while each open point is the volume calculated from the mean mammographic diameter. The calculated regression line had a correlation coefficient of $-0.96$ and a slope of $-9 \times 10^{-3}\,cm^3(\log)\,day^{-1}$. This indicates statistically significant regression ($P < 0.0001$, Student's *t*-test).

*Side-effects*

Side-effects of therapy were assessed at weekly clinical interview. The morbidity associated with cytotoxic therapy was reported using the WHO toxicity grading system (Miller *et al.*, 1981).

*Definitive locoregional surgery*

On completion of systemic therapy (3–6 months) mastectomy with extensive skin removal and axillary node clearance was performed in 82 patients, of whom 55 had simultaneous reconstruction by latissimus dorsi myocutaneous flaps. In six patients with complete clinical response, wide local excision of the previous tumour site was preferred; this being followed by radiotherapy in five cases. The excised specimen was submitted to histological examination.

Patients who had shown a significant response to preoperative endocrine therapy were continued on endocrine therapy following definitive locoregional surgery. Premenopausal patients proceded to oophorectomy, postmenopausal patients received tamoxifen at a daily dose of 20 mg. Further cytotoxic therapy was not given to any patient after surgery.

*Survival*

The follow-up period has been expressed from the time of initiating systemic therapy to the date of analysis. The median period of follow-up was 24 months (range 4–55 months). Locoregional relapse has been defined as recurrence confined to the chest wall, breast or axilla. Supraclavicular lymph node recurrence has been classified as distant metastasis, in keeping with the staging classification for disease at initial presentation (International Union Against Cancer, 1987 TMN classification).

### Results

*Response to endocrine therapy*

Twenty-four of the 61 (39%) patients treated by initial endocrine therapy had significant regression of their tumours (Table III). All responding tumours had an ER concentration of $\geqslant$20 fmol mg cytosol protein$^{-1}$. The proportion of patients achieving regression did not vary greatly in relation to the type of endocrine therapy received or menopausal status and for the purpose of this report all patients receiving endocrine treatment have been considered together.

Of those patients responding to endocrine therapy, the median time taken to achieve half volume (T1/2) was 44 days (range 3–150 days, Figure 2). Only one tumour showed complete clinical regression within the period of the study; however all had residual invasive carcinoma detected by histopathological examination. For various reasons the duration of preoperative antioestrogen therapy was prolonged beyond 12 weeks in eight patients. Seven tumours continued to regress at the same rate while one tumour regarded as static underwent a rapid reduction in size at 5 months.

*Response to cytotoxic therapy*

A significant reduction in tumour volume was observed in 34 of the 47 patients (72%) who received cytotoxic therapy (Table II). Thirteen patients (27.6%) had complete clinical regression of their tumour and eight (17%) had no histological evidence of invasive carcinoma in their mastectomy or wide local excision specimens; five patients had no gross residual disease but invasive carcinoma was visible microscopically. No patient showed evidence of tumour progression during treatment with chemotherapy.

The rate of regression was, on average, more rapid than that achieved with endocrine therapy (median T1/2 of 20 days, range 3–77 days, Figure 2). Those patients with steeper regression slopes were more likely to achieve complete clinical response within the time scale of the study.

There was no significant relationship between significant regression to chemotherapy and the pretreatment variables of age, menopausal status, initial clinical tumour size or pathological axillary node status (Table IV). Of those who failed to respond to endocrine therapy, chemotherapy was more likely to achieve regression in ER-poor tumours (Table IV).

**Table I** Relationship between responses to hormonal therapy and oestrogen receptor concentration of the primary tumour as determined by the dextram-coated charcoal adsorption method in 61 patients with large operable breast cancer

| | *No. of patients with significant regression/total* | | |
|---|---|---|---|
| | *ER <20[a]* | *ER >20[a]* | *Total* |
| Premenopausal | | | |
| Oophorectomy | 0/2 | 2/3 | 2/5 |
| Goserelin | 0/3 | 7/13 | 7/16 |
| Postmenopausal | | | |
| Tamoxifen | 0/5 | 4/6 | 4/11 |
| Aminoglutethimide | 0/4 | 4/6 | 4/10 |
| 4-hydroxyandrostenedione | – | 7/16 | 7/16 |
| Goserelin | 0/1 | 0/2 | 0/3 |
| Total | 0/15 | 24/46 | 24/61 |

[a]fmol mg cytosol protein$^{-1}$.

**Figure 2** Graph illustrating the difference in the time taken to achieve half volume (T1/2) in tumours which responded to endocrine therapy ($n = 24$) and cytotoxic therapy ($n = 34$). The median T1/2 of tumours responding to endocrine therapy was 44 days (range 3–150 days). The median T1/2 of tumours responding to cytotoxic therapy was 20 days (range 3–77 days).

**Table II** Response rates in 47 patients with large operable cancers of the breast treated with four cycles of the chemotherapeutic regime CHOP (cyclophosphamide 1 g m$^{-2}$, adriamycin 50 mg m$^{-2}$, vincristine 1.4 mg m$^{-2}$ to a maximum of 2 g, all by i.v. bolus and oral prednisolone 40 mg per day for 5 days) before definitive locoregional surgery. The $\chi^2$ test has been used to compare the proportion responding to chemotherapy following failed endocrine therapy in relation to ER concentration

| | *No. with significant regression/total* | *No. with complete clinical regression* |
|---|---|---|
| Primary cytotoxic therapy | | |
| ER <20[a] | 23/27 | 8 |
| Following failed endocrine therapy | | |
| ER <20[a] | 8/10 | 4 |
| ER ⩾20[a] | 3/10[b] | 1 |
| Total | 34/47 | 13 |

[a]fmol mg cytosol protein$^{-1}$, [b]statistically significant $\chi_1{}^2 = 5.05$, $P = 0.025$.

**Table III** Relationship between the pretreatment ER concentration and response to 12 weeks endocrine therapy in 61 patients with large operable cancers of the breast. The ER concentration was determined by the dextran-coated charcoal adsorption method

| *ER status* | *Total no.* | *Significant regression* | *No change* | *Progression* |
|---|---|---|---|---|
| ER – poor 20[a] | 15 | 0 | 5 | 10 |
| ER – rich ⩾20[a] | 46 | 24 | 16 | 6 |

[a]fmol mg cytosol protein$^{-1}$.

*Survival and preoperative therapy*

The overall, distant disease-free and disease-free survival of all patients within the study is shown in Figure 3. Local recurrence-free survival is shown in Figure 4. With a median follow-up of 23 months (range 4–55 months), 18 (20%) patients have relapsed, seven of whom have died as a result of their disease. Of these seven patients, three had shown failed to achieve significant regression in response to systemic therapy. In five patients relapse was locoregional alone, six patients had distant metastasis alone while 13 had evidence of both. At 3 years 86% (76–96%; 95% confidence limits) remain alive and 67% (55–79%; 95% confidence limits) remain disease-free, with 81% (70–92%); 95% confidence limits) having no evidence of local recurrence.

*Toxicity*

The side-effects experienced with endocrine treatment were minimal. Hot flushings were noted in seven patients (37%) receiving goserelin but were only moderately severe in two patients. Vaginal dryness was a problem in one patient. Of the 16 patients who received 4-hyroxyandrostenedione

**Table IV** Relationship between pretreatment variables and significant tumour regression following four cycles of preoperative cytotoxic therapy (cyclophosphamide 1 g m$^{-2}$, adriamycin 50 mg m$^{-2}$, vincristine 1.4 mg m$^{-2}$ to a maximum of 2 g, all by i.v. bolus and oral prednisolone 40 mg per day for 5 days). Patients were designated postmenopausal if more than 1 year had elapsed since their last menstrual period. Pretreatment axillary node status as determined from histological examination fo an axillary node sample was available for 29 patients. The $\chi^2$ test has been used to compare the relationship between pretreatment variables and the proportion within each group who achieved significant regression

| | *No. significant regression/total no.* | $\chi^2$ | *P value* |
|---|---|---|---|
| Tumour diameter | | | |
| <5 cm | 19/26 | | |
| 5–<6 cm | 10/14 | 0.813 | 0.666 |
| ≥6 cm | 7/8 | | |
| Age | | | |
| 30–39 years | 3/5 | | |
| 40–49 years | 14/18 | | |
| 50–59 years | 13/19 | 0.939 | 0.816 |
| >60 years | 4/5 | | |
| Menstrual status | | | |
| Premenopausal | 19/25 | | |
| Postmenopausal | 15/22 | 0.357 | 0.55 |
| Oestrogen receptor concentration | | | |
| <20 fmol mg cytosol protein$^{-1}$ | 31/37 | | |
| ≥20 fmol mg cytosol protein$^{-1}$ | 3/10 | 11.38 | 0.0007 |
| Axillary lymph node status | | | |
| Metastasis | 15/23 | | |
| No metastasis | 6/6 | 2.88 | 0.09 |
| Unavailable | 5/13 | | |

**Figure 3** Overall, distant disease-free and relapse-free survival for 88 patients with large operable breast cancer treated by primary systemic therapy before definitive locoregional surgery. The median period of follow-up was 24 months (range 4–55 months).

**Figure 4** Cumulative proportion remaining free from local relapse in 88 patients with operable breast cancers of greater than 4 cm at diagnosis treated with primary systemic therapy before definitive locoregional surgery. The median period of follow-up was 24 months (range 4–55 months).

adverse effects included a tender lump at the injection site ($n = 4$), hot flushings ($n = 2$), erythematous rash on buttocks ($n = 1$), and a clinically insignificant, self-limiting abnormality of liver function tests ($n = 2$). Four patients who received aminoglutethimide complained of nausea and lethargy on initiation of therapy.

The chemotherapeutic regime was moderately toxic. The principle side-effects were alopecia (100%), nausea and vomiting (91% WHO grade 2 or greater), stomatitis (68% WHO grade 2), dyspepsia (20%) mild dysuria (13%) and neutropenia (26% WHO grade 2 or greater). Premature termination of therapy was required in two patients because of nonspecific toxicity and in one on account of iliofemoral thrombosis. There were no treatment related deaths and no increase in morbidity associated with definitive surgery.

### *Axillary lymph node status*

Histological assessment of axillary lymph node status was performed in 46 patients (51%) before and in 86 patients (98%) on completion of systemic therapy. Metastatic carcinoma was detected in 33 (72%) and 42 cases (49%) respectively. Overall 56 patients (64%) had axillary metastases detectable at some stage in their management.

Comparison of pre- and post-treatment axillary node status was possible in 43 patients. Of the 33 patients in whom axillary node metastasis were detected pretreatment, 14 had no evidence of metastases following systemic therapy. Of these eight had shown significant regression during systemic therapy of which five were clinically complete. Axillary node metastases were found in only one of the ten patients in whom the pretreatment axillary node sample had failed to demonstrate metastases. This patient did not respond to 4-hydroxyandrostenedione or proceed to chemotherapy and so may represent a true progression of axillary node status.

### Discussion

This study was undertaken to ascertain whether appropriate long-term systemic therapy could be selected by direct assessment of primary tumour response before surgical excision. Experience of this novel form of management in 88 cases has shown it to be a feasible approach.

A biopsy of the tumour was performed prior to initiation of systemic therapy and has allowed direct correlation of oestrogen receptor concentration to individual tumour response. Of the first 36 patients treated by primary endocrine therapy, no patient with a tumour of ER concentration less than 20 fmol mg cytosol protein$^{-1}$ showed significant regression (Anderson *et al.*, 1989). Thereafter a change in protocol was instituted and primary endocrine therapy was reserved for those patients with tumours of ER concentration $\geqslant$20 fmol mg cytosol protein$^{-1}$. Individual responsiveness to endocrine therapy, even within ER-moderate/rich tumours however could only reliably be determined by direct observation of the effect of therapy. In this way we have selected out those patients in whom continuing endocrine systemic therapy is appropriate. Such patients would appear to have an excellent prognosis (Anderson *et al.*, 1989). Similarly direct objective assessment of tumour response to systemic therapy allows cessation of endocrine therapy where it has been demonstrated to be of no value with initiation of chemotherapy if desired.

A variety of endocrine therapies have now been tried and the proportion of patients achieving regression is similar to that documented using the same agents in advanced disease (Hawkins, 1985; Coombes, Stein & Dowsett, 1989; Nicholson & Waler, 1989). Of particular interest is the efficacy of the gonadotrophin-releasing hormone agonist, goserelin and the peripheral aromatase inhibitor 4-hydroxandrostenedione in pre- and post-menopausal women respectively. Gonadotrophin-releasing hormone agonists produce an effect similar to oophorectomy but without operation (Nicholson & Walker, 1989) and would appear to be suitable for the primary treatment of premenopausal women. Furthermore they can be discontinued should therapy prove ineffective. The requirement for intramuscular injection of 4-hydroxyandrostenedione is a disadvantage and tamoxifen is preferred for primary therapy in postmenopausal women.

Within this study cytotoxic therapy with its greater associated toxicity was reserved for patients in whom endocrine therapy had failed or the likelihood of response to endocrine therapy was minimal (i.e. patients with ER-poor or ER-negative tumours). The proportion of such patients with tumours which were chemosensitive was high and lies within the observed range of 70–93% described with 'neoadjuvant' chemotherapy in more locally advanced breast cancers (Jacquillat *et al.*, 1988; Hortobagyi *et al.*, 1988; Swain *et al.*, 1987). Of those individual tumours directly demonstrated as endocrine-resistant however the proportion of ER-poor/negative tumours regressing with chemotherapy paralleled that of primary chemotherapy ($\sim$80%) but the efficacy in ER-rich tumours was much lower (30%). This difference in response pattern may suggest a common mechanism of failure to respond to both endocrine and cytotoxic therapies.

The response to endocrine therapy was on average, slower than that achieved with cytotoxic therapy. Within the period of the study only one patient achieved complete clinical regression of their tumour during endocrine therapy and in no patient was pathological remission complete. In contrast the response to cytotoxic therapy was occasionally rapid and in those patients with such a rapid response, complete clinical and even complete pathological response was observed. None of the parameters studied were able to define which patients were more likely to achieve such complete remission.

As this study has progressed refinements have been made to the protocol. The use of ER data to select systemic therapy has already been described. Initially axillary node sampling was not an integral part of the pretreatment assessment. Analysis of the pathological post-treatment axillary node staging of the first 43 patients demonstrated a lower incidence (51%) of positivity than would be expected. Since clinical axillary node staging is notoriously unreliable it was felt that pathological staging of the axillary nodes should be included in the preoperative assessment if survival data was to be assessed. Of the 45 patients in whom pretreatment axillary node status was known there was a higher incidence of lymph node metastases (73%) which is more in keeping with previous studies for tumours of similar stage (Carter *et al.*, 1989). Following therapy however only 20 (44%) had detectable node metastases suggesting that the preliminary figure of 51% was not due to sample bias. While it is conceivable that axillary node sampling has simply removed the few lymph nodes with metastatic disease it is also possible that the systemic therapy has been effective in eradicating axillary metastases.

A possible benefit of primary systemic therapy, which we have not yet explored, is that it may permit conservative surgery in patients with large tumours which would otherwise require mastectomy. The usefulness of initial systemic therapy with the aim of avoiding mastectomy has been reported in a series of 57 patients with large but potentially operable breast cancers (Mansi *et al.*, 1989). With a median follow-up of 19 months these authors report similar response rates, loco-regional recurrence, distant relapse and projected overall survival rates to this study with only 18% (10/57) of patients subsequently proceeding to mastectomy. Primary systemic therapy however is not yet orthodox for operable disease and we did not believe it justified to perform less than a mastectomy in the majority of patients. In future studies a more conservative approach would be worthy of trial.

A disadvantage of preoperative systemic therapy is the potential psychological morbidity induced by leaving the tumour *in situ* while initial systemic therapy is undertaken. In general this did not prove to be a problem even in patients with nonresponsive disease. This was probably due to the fact that surgical removal of the tumour, still regarded by many patients as the critical step in their managment, was still possible. Formal examination of psychological morbidity was not however undertaken during this study, but should be part of any future work.

In addition to the benefit of selecting appropriate systemic therapy, there is theoretical (Goldie & Coldman, 1979; Skipper 1964), experimental (Fisher *et al.*, 1983) and clinical (Nissen-Meyer *et al.*, 1986; Ragaz, 1986) evidence, that early administration of systemic therapy in the treatment schedule of patients with breast cancer may improve survival. The 3 year cumulative survival rate within this study was 86%, with 81% of patients remaining free of local recurrence. This compares favourably to 3 year survival rates achieved with orthodox treatment of large tumours of similar stage which range from 65–78% (Duncan & Kerr, 1976; Sorace & Lippman, 1988; Carter *et al.*, 1989). Proof requires a controlled randomised trial in which this selective approach is assessed against conventional treatment, that is mastectomy followed by combination chemotherapy in premenopausal women and tamoxifen in postmenopausal women; such a trial is currently underway in Edinburgh.

We are grateful to the staff at the Breast Unit, Longmore Hospital for their care and consideration of these patients, Mr D. Carston and Miss A. Tesdale (University Department of Surgery, University of Edinburgh) who performed the oestrogen receptor assays, the staff of the University Department of Pathology for processing and handling of biopsy specimens and to Professor D.C. Carter (University Department of Surgery, University of Edinburgh) and Professor J. Smyth (University Department Clinical Oncology) for their continuing support in this project. We thank ICI for their supply of goserelin and Ciba Geigy for providing the intramuscular preparation of 4-hydroxyandrostenedione. The study was supported by the Cancer Research Campaign and approved by the Hospital Ethical Committee.

### References

ANDERSON, E.D.C., FORREST, A.P.M., LEVACK, P.A., CHETTY, U. & HAWKINS, R.A. (1989). Response to endocrine manipulation and oestrogen receptor concentration in large operable breast cancer. *Br. J. Cancer*, **60**, 223.

BIANCO, A.R., DE PLACIDO, S., GALLO, C. & 5 others (1988). Adjuvant therapy with tamoxifen in operable breast cancer. 10 year results of Naples (Gun) study. *Lancet*, **ii**, 1095.

CARTER, C.L., ALLEN, C. & HENSON, D.E. (1989). Relation of tumour size, lymph node status and survival in 24,740 breast cancer cases. *Cancer*, **63**, 181.

COOMBES, R.C., STEIN, R.C. & DOWSETT, M. (1989). Aromatase inhibitors in human breast cancer. *Proc. Royal Soc. Edin.*, **95B**, 283.

DUNCAN, W. & KERR, G.R. (1976). The curability of breast cancer. *Br. Med. J.*, **2**, 781.

EARLY BREAST CANCER TRIALISTS' COLLABORATIVE GROUP (1988). Effects of adjuvant tamoxifen and of cytotoxic therapy on mortality in early breast cancer. An overview of 61 randomised trials among 28,896 women. *N. Engl. J. Med.*, **319**, 1681.

FISHER, B., GUNDEZ, N. & SAFFER, E.A. (1983). Influence of the interval between primary tumour removal and chemotherapy on kinetics and growth of metastases. *Cancer Res.*, **43**, 1488.

FISHER, B., REDMAN, C., BROWN, A. & 8 others (1986). Adjuvant chemotherapy with and without tamoxifen in the treatment of primary breast cancer: 5-year results from the National Surgical Adjuvant Breast and Bowel Projecy Trial. *J. Clin. Oncol.*, **4**, 459.

FORREST, A.P.M., LEVACK, P.A., CHETTY, U. & 4 others (1986). A human tumour model. *Lancet*, **ii**, 840.

GLASS, A., WEIAND, H.S., FISHER, B. & 8 others (1981). Acute toxicity during adjuvant chemotherapy for breast cancer: The National Surgical Adjuvant Breast and Bowel Project (NSABP) experience from 1717 patients receiving single and multiple agents. *Cancer Treat. Res.*, **65**, 363.

GOLDIE, J.H. & COLDMAN, A.J. (1979). A mathematic model for relating the drug sensitivity of tumours to their spontaneous mutation rate. *Cancer Treat. Rep.*, **63**, 1727.

HAWKINS, R.A., HILL, A. & FREEDMAN, B. (1975). A simple method for the determination of oestrogen receptor concentrations in breast tumours and other tissues. *Clin. Chim. Acta*, **64**, 203.

HAWKINS, R.A. (1985). Receptor assays and the clinical management of breast cancer. *Scott. Med. J.*, **30**, 73.

HAWKINS, R.A., BLACK, R., STEELE, R.J.C., DIXON, J.M.J. & FORREST, A.P.M. (1981). Oestrogen receptor concentration in primary breast cancer and axillary node metastases. *Breast Cancer Res. Treat.*, **1**, 245.

HORTOBAGYI, G.N., AMES, G.R., BUZDAR, F.C. & 7 others (1988). Management of stage III primary breast cancer with primary chemotherapy, surgery, and radiation therapy. *Cancer*, **62**, 2507.

INTERNATIONAL UNION AGAINST CANCER (1987). *TNM Classification of Malignant Tumours*. Hermanek, P., Sobin, L.H. (eds), 4th fully revised edition. Springer-Verlag: Berlin, p. 93.

JACQUILLAT, C.L., BAILLET, F., WEIL, M. & 8 others (1988). Results of a conservative treatment combining induction (neoadjuvant) and consolidation chemotherapy, hormonotherapy, and external and interstitial irradiation in 98 patients with locally advanced breast cancer (IIIA–IIIB). *Cancer*, **61**, 1977.

MANSI, J.L., SMITH, I.A., WALSH, G. & 6 others (1989). Primary medical treatment for operable breast cancer. *Eur. J. Clin. Oncol.*, **25**, 1623.

MILLER, A.B., HOOGSTRATEN, B., STRAQUET, M. & WINKLER, A. (1981). Reporting results of cancer treatment. *Cancer*, **147**, 207.

NICHOLSON, R.I. & WALKER, K.J. (1989). Use of LH-RH agonists in the treatment of breast disease. *Proc. Royal Soc. Edin.*, **95B**, 271.

NISSEN-MEYER, R., HOST, H., KJELLGREN, K., MANSSON, B. & BORIN, T. (1986). Perioperative adjuvant chemotherapy of breast cancer: The Scandinavian experience. *Recent Results in Cancer Research*, **103**, 95.

NOLVADEX ADJUVANT TRIAL ORGANISATION (1988). Controlled trial of tamoxifen as single adjuvant agent in management of early breast cancer. Analysis at eight years. *Br. J. Cancer*, **57**, 608.

PALSHOF, T., CARSTENSENEN, B., MOURIDSEN, H.T. & DOMBERNOWSKY, P.(1985). Adjuvant endocrine therapy in pre- and postmenopausal women with operable breast cancer. *Reviews on Endocrine-related Cancer*, Suppl. 17, 43.

RAGAZ, J. (1986). Preoperative (neoadjuvant) chemotherapy for breast cancer: outline of the British Columbia Trial. *Recent Results Cancer Res.*, **103**, 85.

ROSE, C., THORPE, S.M., ANDERSEN, K.W. & 4 others (1985). Beneficial effect of adjuvant tamoxifen therapy in primary breast cancer patients with high oestrogen receptor values. *Lancet*, **i**, 16.

SCOTTISH CANCER TRIALS OFFICE (M.R.C.) EDINBURGH (1987). Adjuvant tamoxifen in the management of operable breast cancer: the Scottish Trial Report from the Breast Cancer Trials Committee. *Lancet*, **ii**, 171.

SKIPPER, H.E., SCHABEL, F.M Jr & WILCOX, W.S. (1964). Experimental evaluation of potential anticancer agents XIII on the criteria and kinetics associated with 'curability' of experimental leukemia. *Cancer Chem.* Rep. 35, 1.

SORACE, R.A. & LIPPMAN, M.E. (1988). Locally advanced breast cancer. In *Diagnosis and Management of Breast Cancer*, Lippman, M.E., Lichter, A.S. & Danford, D.N. (eds) p. 272. Sanders, W.B.: Philadelphia.

SWAIN, S.A., SORACE, R.A., BAGLEY, C.S. & 5 others (1987). Neoadjuvant chemotherapy in the combined modality approach of locally advanced nonmetastatic breast cancer. *Cancer Res.*, **47**, 3889.